# *Mycobacterium leprae*-Infected Macrophages Preferentially Primed Regulatory T Cell Responses and Was Associated with Lepromatous Leprosy

**DOI:** 10.1371/journal.pntd.0004335

**Published:** 2016-01-11

**Authors:** Degang Yang, Tiejun Shui, Jake W. Miranda, Danny J. Gilson, Zhengyu Song, Jia Chen, Chao Shi, Jianyu Zhu, Jun Yang, Zhichun Jing

**Affiliations:** 1 Shanghai Dermatology Hospital, Shanghai, P. R. China; 2 Yunnan Center for Disease Control and Prevention, Kunming, P. R. China; 3 Ian Therapeutics and Research Lab, Vancouver, British Columbia, Canada; 4 BGC Biotechnology Research Center, Shandong, P. R. China; Hospital Infantil de Mexico Federico Gomez, UNITED STATES

## Abstract

**Background:**

The persistence of *Mycobacterium leprae* (*M*. *leprae*) infection is largely dependent on the types of host immune responses being induced. Macrophage, a crucial modulator of innate and adaptive immune responses, could be directly infected by *M*. *leprae*. We therefore postulated that *M*. *leprae*-infected macrophages might have altered immune functions.

**Methodology/Principal Findings:**

Here, we treated monocyte-derived macrophages with live or killed *M*. *leprae*, and examined their activation status and antigen presentation. We found that macrophages treated with live *M*. *leprae* showed committed M2-like function, with decreased interleukin 1 beta (IL-1beta), IL-6, tumor necrosis factor alpha (TNF-alpha) and MHC class II molecule expression and elevated IL-10 and CD163 expression. When incubating with naive T cells, macrophages treated with live *M*. *leprae* preferentially primed regulatory T (Treg) cell responses with elevated FoxP3 and IL-10 expression, while interferon gamma (IFN-gamma) expression and CD8^+^ T cell cytotoxicity were reduced. Chromium release assay also found that live *M*. *leprae*-treated macrophages were more resistant to CD8^+^ T cell-mediated cytotoxicity than sonicated *M*. *leprae*-treated monocytes. *Ex vivo* studies showed that the phenotype and function of monocytes and macrophages had clear differences between L-lep and T-lep patients, consistent with the *in vitro* findings.

**Conclusions/Significance:**

Together, our data demonstrate that *M*. *leprae* could utilize infected macrophages by two mechanisms: firstly, *M*. *leprae*-infected macrophages preferentially primed Treg but not Th1 or cytotoxic T cell responses; secondly, *M*. *leprae*-infected macrophages were more effective at evading CD8^+^ T cell-mediated cytotoxicity.

## Author Summary

Macrophages play critical roles in modulating *Mycobacterium leprae* (M. leprae) infection. In this study, we investigated the immune status of macrophages stimulated with live *M*. *leprae* or killed *M*. *leprae*. We found that live *M*. *leprae*-infected macrophages polarized toward the regulatory M2-type, preferentially primed Treg responses, and downregulated Th1 and cytotoxic T cell responses. Furthermore, our *ex vivo* data confirmed these findings. These results revealed novel mechanisms of *M*. *leprae* infection.

## Introduction

The ability of an intracellular pathogen to establish a productive infection relies on its ability to evade cytotoxic T cell-mediated clearance of infected cells. In the case of *Mycobacterium leprae* (*M*. *leprae*), an obligate intracellular pathogen that is dependent on the host fatty acid metabolism for microbial lipid synthesis[1], the outcome of *M*. *leprae*-caused leprosy is strongly associated with the types of immune responses being activated[2]. At one end of the spectrum, the lepromatous leprosy (L-lep) is a progressive disease with numerous lesions, plenty of intracellular bacteria, and is associated with weak or absent cellular immunity and increased FoxP3^+^ T cells at lesion site[3–6]. In contrast, the tuberculoid leprosy (T-lep) at the other end of the spectrum is a self-contained disease with fewer lesions, low or undetectable intracellular bacteria, and is associated with robust Th1-skewing antigen-specific cellular immunity[7,8]. Therefore, it is believed that host immune systems dictate the clinical outcome of *M*. *leprae* infections.

Monocytes and monocyte-derived macrophages are important antigen-presenting cells and are crucial in stimulating and shaping the adaptive immune responses. The state of macrophage activation, be it proinflammatory (M1-type) or anti-inflammatory (M2-type), can directly modulate the surrounding microenvironment and influence the types of T cell activation and differentiation[9]. Activation of M1-type macrophages is associated with the presence of interferon gamma (IFN-gamma), a main Th1 product, and results in increased MHC class II and tumor necrosis factor (TNF), interleukin 1 (IL-1), IL-6, and IL-12 expressions[10,11]. On the other hand, M2-type macrophages can be activated by IL-4, IL-13, and/or IL-10 stimulation and were thought to induce regulatory responses through IL-10 production, with downregulated MHC class II and upregulated CD163 expression. Relevant to *M*. *leprae* infection and leprosy, M1-type macrophages could induce killing of *Mycobacterium* through nitric oxide release and promote Th1-type immunity[12], while IL-10-producing M2-type macrophages subverted the Th1 response[13]. An enrichment of M2 genes and expression of CD163 were observed in L-lep lesions but not in T-lep lesions[14,15]. Macrophages are also an infection target of *M*. *leprae*, which could potentially alter the activation status of infected macrophages. How *M*. *leprae*-infection influences the T cell priming function of infected macrophages is currently not completely studied.

Though enrichment of FoxP3^+^ T cells and M2-type macrophages was previously observed in L-lep, in contrast to T-lep, it is yet unclear whether preexisting immunosppression mechanisms in an individual tended to lead to a more severe disease, or *M*. *leprae*-infection could actively subvert the immune response toward a more regulatory type. The previous observation that live bacillus Calmette-Guerin (BCG) vaccine, made from *M*. *bovis*, induced immunosuppressive T cell phenotype and function when added into peripheral blood mononuclear cells (PBMCs) led to our postulation that live infection of macrophages by *M*. *leprae* may alter the antigen presentation and T cell priming function of infected macrophages[16]. We examined this possibility in this study.

## Materials and Methods

### Ethics statement

The Shanghai Dermatology Hospital Institutional Animal Care and Use Committee approved the animal procedures (protocol: 3396). All animals were cared for in accordance with the guidelines of the Committee on Care and Use of Laboratory Animals (Institute of Laboratory Animal Resources, National Research Council). Human specimens were used according to the guidelines approved by the Ethical Committee of Shanghai Dermatology Hospital (No. 125988). All participants provided written informed consent.

### Study subjects

Leprosy patients were classified according to the criteria of Ridley and Jopling[3], and age- and sex-matched healthy volunteers were recruited (Table 1). Peripheral blood samples were obtained from all participants by venipuncture, and skin biopsy specimens (6 mm in diameter) were collected by standard punch technique from agreeing participants. All participants were recruited in Shanghai Dermatology Hospital. Patients with clinically significant autoimmune diseases or other serious diseases such as tumor, cardiovascular diseases, diabetes and chronic hepatitis were excluded.

**Table 1:** Study subject information of healthy volunteers, L-lep patients and T-lep patients.

	Healthy	L-lep	T-lep	*p*
N	6	13	12	
Sex (F/M)	4/2	9/4	8/4	> 0.05
Age, y	35–65	44–68	47–71	> 0.05

### Cell isolation

For isolation of blood immune cells, PBMCs were first obtained by collecting buffy coat from Ficoll-Paque centrifugation, and were cryopreserved in -80°C for less than 1 year. Monocytes were then purified by using Human Monocyte Isolation Kit II (Miltenyi) with purity > 96%. Naive T cells were purified by using Naive Pan T Cell Isolation Kit (Miltenyi) on PBMCs. Purity of naive T cells were confirmed by CD3^+^CD45RA^+^ staining and was > 94%. Total T cells and CD8^+^ T cells were purified by using Pan T Cell Isolation Kit and CD8^+^ T Cell Isolation Kit (Miltenyi) on T cell-monocyte coculture, respectively. For isolation of lesion macrophages, a protocol was adapted from a previously published method on isolating human intestinal macrophages[17], with >95% viability by propidium iodide staining. Macrophage identity was confirmed by microscopic examination and was used fresh. For deriving macrophages in vitro, 10^6^ per mL purified blood monocytes were cultured in RPMI 1640 supplemented with L-glutamine, Pen Strep (Invitrogen) and 10% autologous serum for 6 days in a 6-well plate, at 37°C 5% CO_2_. Media was replaced every 2 days. By day 6, the plate was shaken gently and the upper level media were carefully removed such that only live adherent macrophages remained.

### Culture with *M*. *leprae*

Live *M*. *leprae* (Thai-53 strain) was propagated in athymic BALB/c-nu/nu mice and was harvested from the mouse footpad after 9 months using previously described methods[18,19], and then thawed and washed in PBS/0.05% Tween 80 (Sigma-Aldrich)[20,21]. For heat-killing, bacteria were inactivated at 80°C for 30 min. In vitro derived macrophages or naive T cells were incubated with *M*. *leprae* at a multiplicity of infection (MOI) of 20 for 72 hours. Excess bacteria were then removed by washing the cells twice in culture media.

### Coculture with T cells

Macrophages after *M*. *leprae* incubation were cultured with purified naive T cells at 1-to-1 ratio for 6 days in plain culture media (RPMI 1640 supplemented with L-glutamin, Pen Strep, and 10% FCS). Total T cells or CD8^+^ T cells were then purified and cultured alone in culture media supplemented with 1μg/ml anti-CD3 antibody (BD) and anti-CD28 antibody (eBioscience) for 72 hours.

### Luminex and ELISA

For Luminex assay, 2×10^5^ cells were incubated at the bottom and cytokine-capture beads (Novex) incubated at the top of a 1μm-pore size 96-well transwell plate (Corning), which allowed the transfer of secreted cytokines but not cells. After 6 days in 37°C and 5% CO_2_, the beads were lifted from the top compartment and were transferred to a reader plate. The cytokine concentrations were then measured by a Luminex assay technician. For ELISA, cells after incubation were spun at 1500 rpm for 5 min. 100μl supernatant was taken per well per 2×10^5^ cells. Human IFN gamma and IL-10 ELISA kits (eBioscience) were used.

### Flow cytometry

To evaluate cell purity and phenotype, cells were incubated with combinations of anti-human CD3 (OKT3), CD4 (RPA-T4), CD8 (HIT8a), CD14 (HCD14), CD16 (3G8), CD45RA (HI100) (BD), HLA-DR (L243) and HLA-DQ (Tu169) (BioLegend) for 30 min at 4°C. After staining, cells were washed with culture media, fixed in 1% paraformaldehyde, and analyzed by flow cytometry. FoxP3 staining was done using FoxP3 antibody (259D/C7) (BD) and FoxP3 Fix/Perm Buffer Set (BioLegend) on surface stained cells following manufacturer’s instructions.

### Chromium release assay

T cell cytotoxicity was measured using a standard 4-hour chromium-51 release assay, using purified CD8^+^ T cells as the effector cell and live *M*. *leprae-infected* macrophage or monocytes loaded with sonicated *M*. *leprae* as the target cell.

### Statistical analysis

All error bars represent standard deviation. Data normality was determined by Shapiro-Wilks test. For datasets that did not distribute normally, Kruskal-Wallis one-way and two-way ANOVA and Dunn’s test was used for data with multiple groups. Mann-Whitney test was used for data with two groups. Wilcoxon matched-pairs test was used for paired comparison. *p* < 0.05 from a two-tailed test is considered statistically significant.

## Results

### Viable *M*. *leprae*-infected macrophages polarized toward the regulatory M2-type

**Fig 1:**
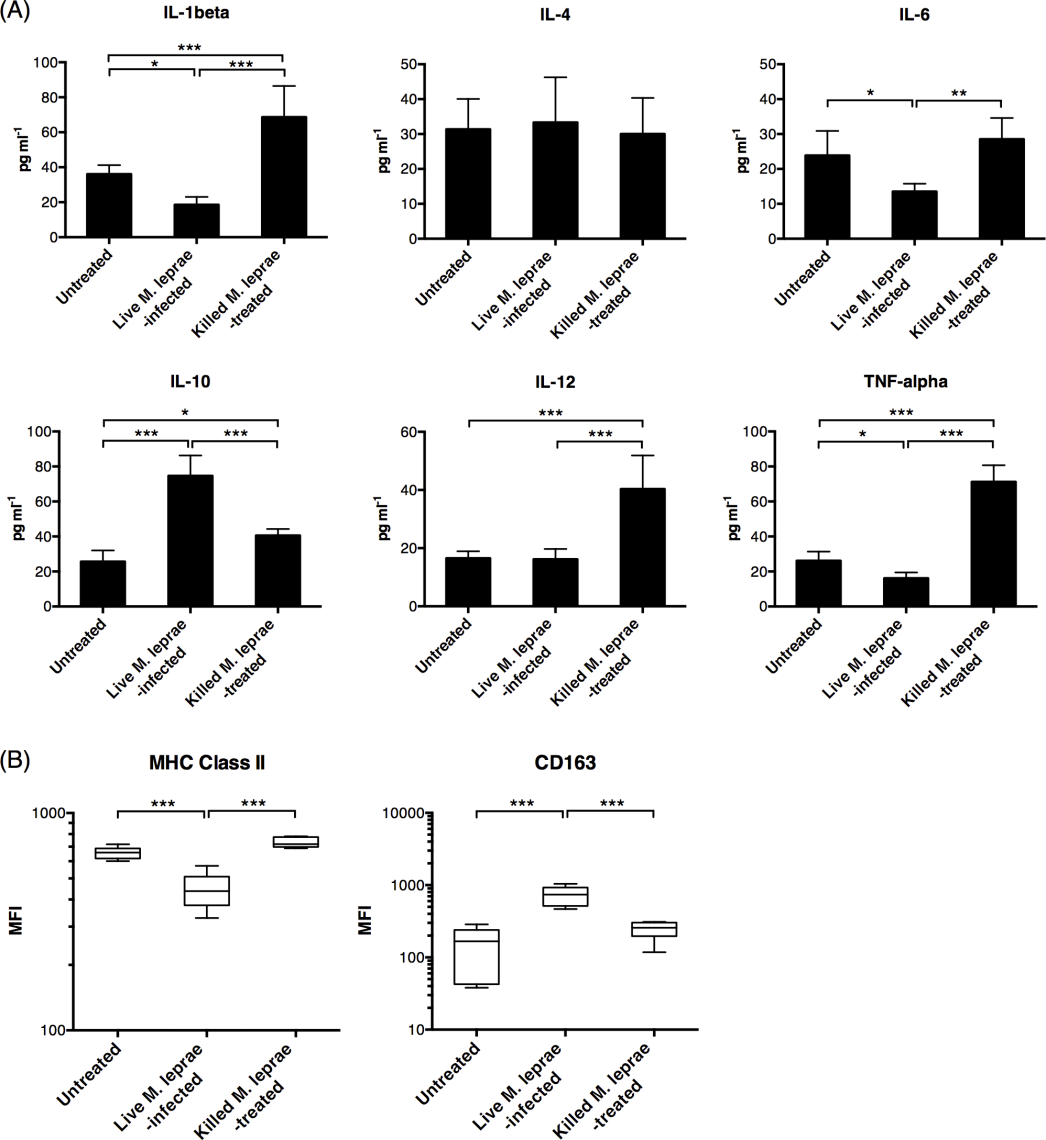
Viable *M*. *leprae* induced M2-type macrophage differentiation. Peripheral blood monocytes were obtained from healthy volunteers and were differentiated into macrophages in vitro. Viable or heat-killed *M*. *leprae* were added to the macrophage culture for 6 days. (A) Cytokine expression by macrophages during coculture, as measured by Luminex assay. N = 6. (B) Mean fluorescence intensity (MFI) of MHC class II and CD163 expression on macrophages after 6-day incubation in all healthy volunteers. N = 6. *: p < 0.05. **: p < 0.01. ***: p < 0.001.

**Fig 2:**
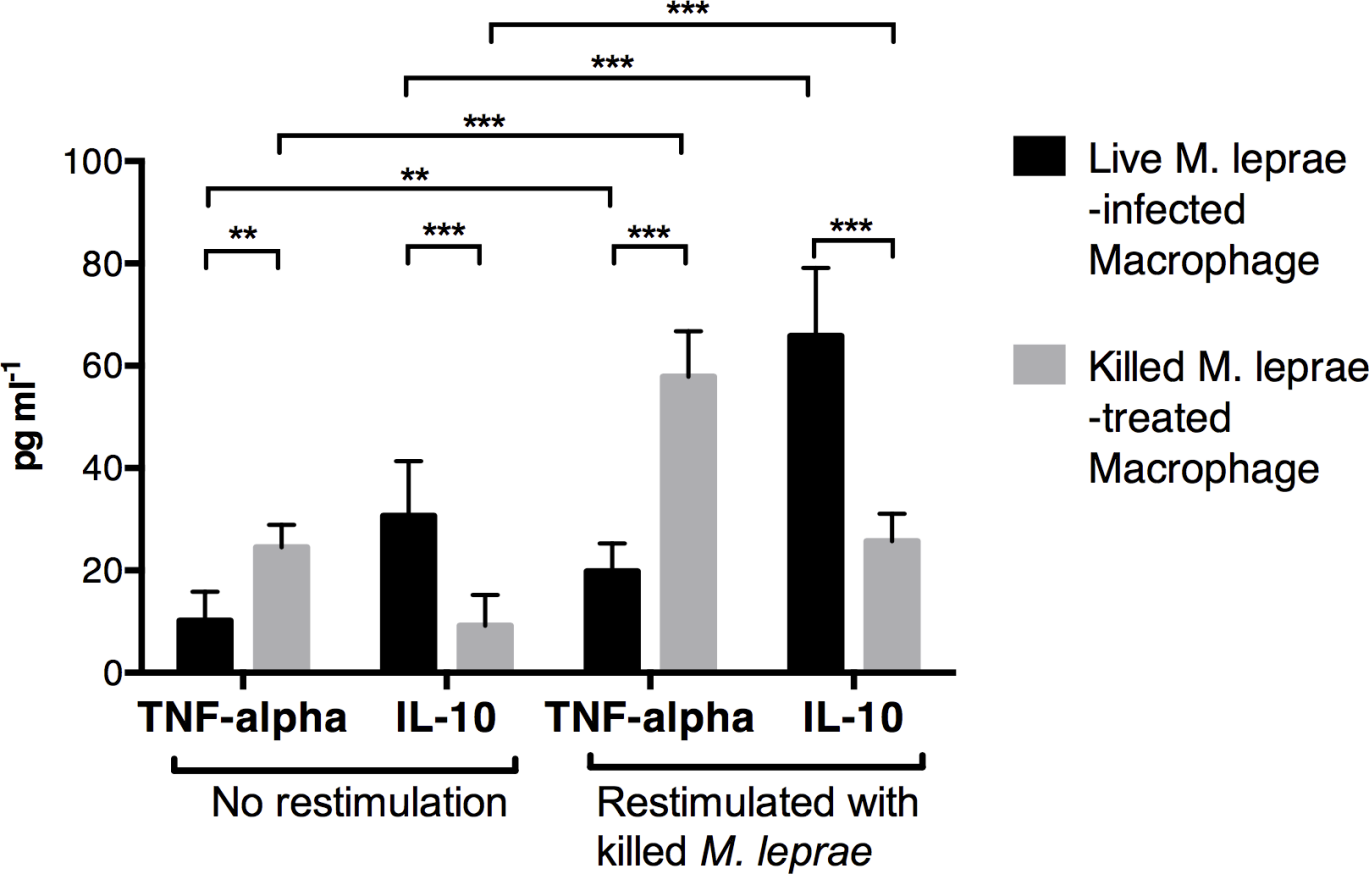
Macrophage treated with different *M*. *leprae* antigens showed committed functional differentiation. Live *M*. *leprae*-infected macrophages or killed *M*. *leprae*-treated macrophages were either restimulated with killed *M*. *leprae*, or cultured in plain culture medium for 6 days. Cytokine expression during the restimulation period was measured by Luminex assay. **: p < 0.01. ***: p < 0.001.

To investigate the effect of *M*. *leprae* infection on antigen presentation, macrophages derived from monocytes isolated from healthy volunteers were cultured with viable *M*. *leprae* for 6 days with autologous serum. These macrophages were hereafter referred to as live *M*. *leprae*-infected macrophages. In a separate culture, heat-killed *M*. *leprae* were added to healthy monocyte derived macrophages (hereafter referred to as killed *M*. *leprae*-treated macrophages). We found that compared to untreated macrophage, live *M*. *leprae*-infected macrophages expressed significantly higher IL-10 in the supernatant, while the expression IL-1beta, TNF-alpha and IL-6 were downregulated (Fig 1A). The expression level of MHC class II molecules (HLA-DR + HLA-DQ) was downregulated, while CD163 was upregulated (Fig 1B). In contrast, killed *M*. *leprae*-treated macrophages expressed significantly higher IL-1beta, IL-12 and TNF-alpha (Fig 1A). The expression of IL-10 by killed *M*. *leprae*-treated macrophages was higher than that by the untreated macrophages but lower than that by the live *M*. *leprae*-infected macrophages (Fig 1A). The heat-killing process would result in bacterial cell rupture and might expose internal *M*. *leprae* antigens, such as bacterial DNA and RNA motifs, that could activate immune responses directly and were not present on live *M*. *leprae*. These internal antigens not seen by macrophages incubated with viable *M*. *leprae* might alone be responsible for IL-1beta, IL-12 and TNF-alpha upregulation. To examine this possibility, the macrophages were first incubated with viable or killed *M*. *leprae* for 6-days, washed twice to remove excess antigens and were then restimulated with killed *M*. *leprae*. We found that restimulation enhanced, rather than suppressed, IL-10 production in live *M*. *leprae*-infected macrophages, in striking contrast to the killed *M*. *leprae*-treated macrophages where an upregulation of TNF-alpha was seen (Fig 2). Collectively, these data demonstrated that the polarization of macrophages after incubation with viable or killed *M*. *leprae* were committed and did not change after restimulation.

### Live *M*. *leprae*-infected macrophages preferentially primed regulatory T cell responses

**Fig 3:**
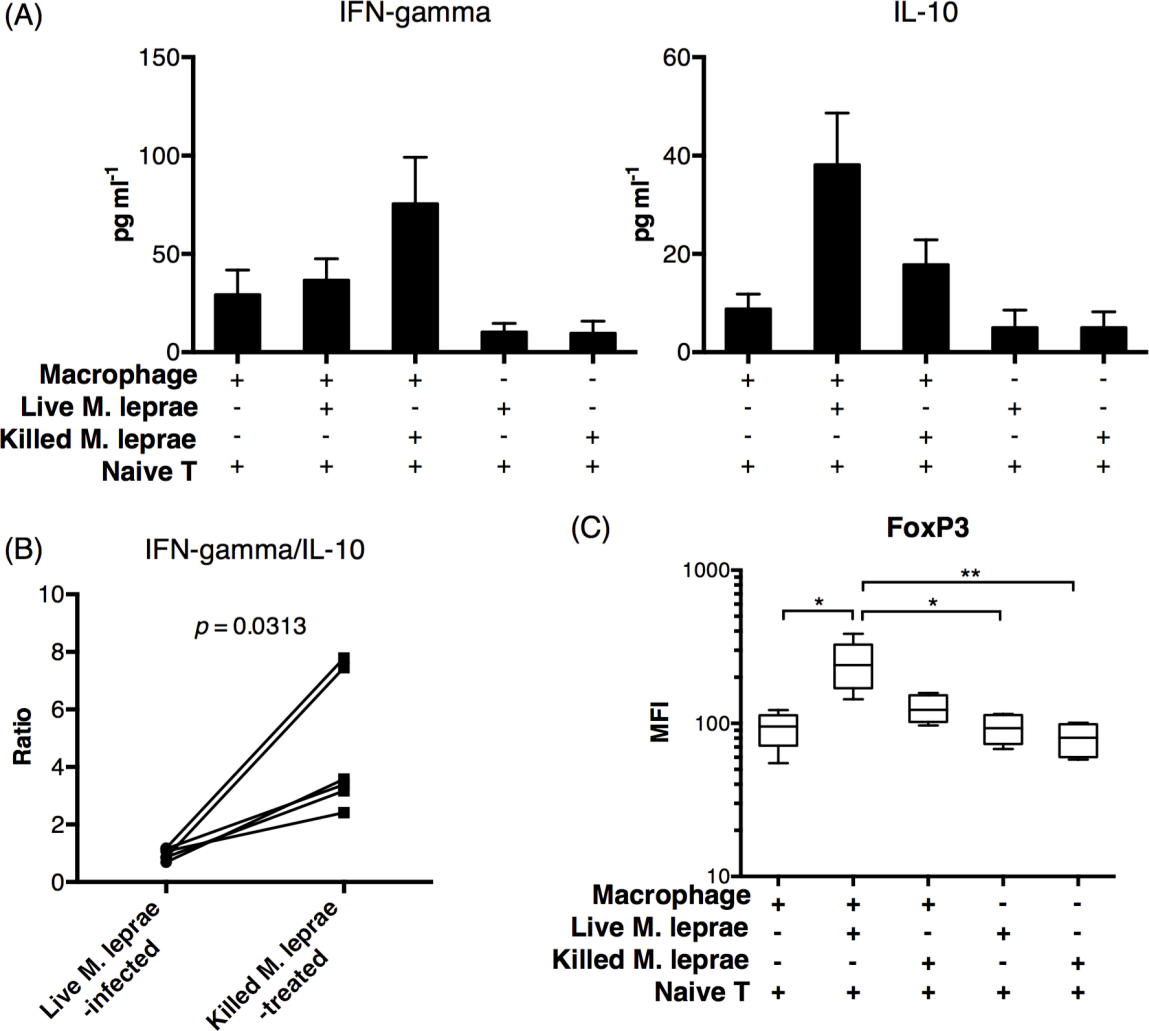
Live *M*. *leprae*-infected macrophages preferentially primed Treg cell responses. Autologous CD45RA^+^ naive T cells were incubated with live *M*. *leprae*-infected macrophages or killed *M*. *leprae*-treated macrophages for 6 days. The T cells were then negatively selected and incubated separately in anti-CD3/CD28-stimulated media for an additional 72 hours, after which the supernatant was collected for ELISA and cells for flow cytometry. (A) Summary of IFN-gamma and IL-10 concentration from all healthy volunteers in the supernatant. N = 6. (B) Ratio of IFN-gamma-to-IL-10 in each individual. (C) Mean fluorescence intensity (MFI) of FoxP3 expression in T cells from all treatment conditions. N = 6. *: p < 0.05. **: p < 0.01.

Macrophage activation and antigen-presentation link the innate and adaptive arms of immunity and play an important role in shaping and remodeling adaptive immunity[22]. It was reported that stimulation of PBMCs with *M*. *bovis*-based live BCG vaccine induced regulatory T cell activity[4,16]. To evaluate the effect of *M*. *leprae* infection on T cell priming, we cocultured CD45RA^+^ naive T cells with untreated macrophages, live *M*. *leprae*-infected macrophages, or killed *M*. *leprae*-treated macrophages for 6 days. At the end of coculture, T cells were negatively purified and restimulated by anti-CD3/CD28. After 72 hours, the cytokine expression by purified T cells was measured by ELISA. We found that T cells incubated with live *M*. *leprae*-infected macrophages preferentially expressed IL-10 while T cells incubated with killed *M*. *leprae*-treated macrophages preferentially expressed IFN-gamma (Fig 3A). Naive T cells incubated directly with viable or killed *M*. *leprae* did not express significant amounts of IFN-gamma or IL-10 (Fig 3A). The IFN-gamma-to-IL-10 ratio was significantly higher in T cells incubated with killed *M*. *leprae*-treated macrophages (Fig 3B). Since IFN-gamma and IL-10 are primarily expressed by Th1 and Treg type cells[23], respectively, we then examined the incubated T cell by flow cytometry. T cells incubated with live *M*. *leprae*-infected macrophages expressed significantly higher frequencies of FoxP3 (Fig 3C), a transcription factor commonly found in Treg cells[24]. Collectively, these data suggest that live *M*. *leprae*-infected macrophages preferentially primed Treg-type responses.

### Reduced cytotoxicity in T cells primed by live *M*. *leprae*-infected macrophages

**Fig 4:**
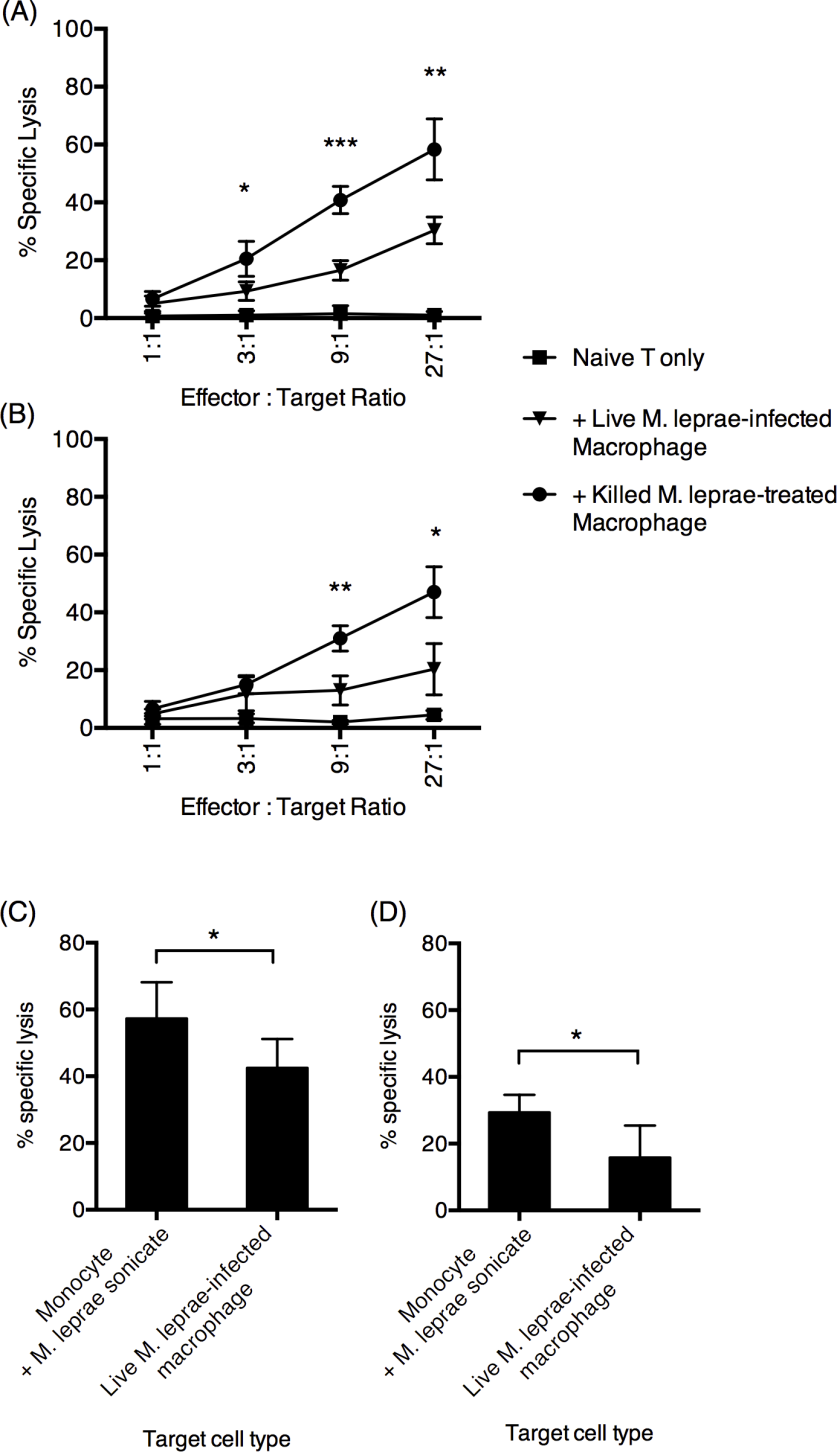
Macrophages incubated with viable *M*. *leprae* suppressed CD8^+^ T cell cytotoxicity. Viable or killed *M*. *leprae* were incubated with T cells for 6 days, after which the CD8^+^ T cells were negatively purified from the coculture and were added to chromium-51-labeled target cells at the indicated effector-to-target ratio. (A) Percentage specific lysis using purified autologous monocyte loaded with sonicated *M*. *leprae* antigen as the target cells. (B) Percentage specific lysis using in vitro derived, *M*. *leprae*-infected macrophages as the target cells. The CD8^+^ T cell-induced cytotoxicity (isolated CD8^+^ T cell + target cell culture minus naive T cell + target cell culture) was shown in (C) and (D), at 27-to-1 effector-to-target ratio. The effectors are (C) CD8^+^ T cells incubated with killed *M*. *leprae*-stimulated macrophages and (D) CD8^+^ T cells incubated with live *M*. *leprae*-infected macrophages. N = 6, with two independent repetitions. *: p < 0.05. **: p < 0.01. ***: p < 0.001.

Cytotoxic T cell-mediated killing of infected cells is responsible for the clearance of intracellular pathogens. We then assessed the cytotoxic capacity of CD8^+^ T cells by a chromium release assay. Freshly isolated chromium-labeled monocytes loaded with sonicated *M*. *leprae* debris were used as the target. CD8^+^ T cells primed by live *M*. *leprae*-infected or killed *M*. *leprae*-treated macrophages were negatively selected and were then added to chromium-labeled, *M*. *leprae* antigen-loaded macrophages. We found that CD8^+^ T cells primed by live *M*. *leprae*-infected macrophages resulted in significantly reduced levels of chromium release, compared to CD8^+^ T cells primed by killed *M*. *leprae*-treated macrophages (Fig 4A). In a separate experiment, we repeated the chromium release assay using live *M*. *leprae*-infected macrophages as the target cell. We found that CD8^+^ T cells primed by killed *M*. *leprae*-treated macrophages was more efficient at killing live *M*. *leprae*-infected macrophages, than CD8^+^ T cells primed by live *M*. *leprae*-infected macrophages (Fig 4B). Moreover, live *M*. *leprae*-infected macrophages were more resistant to CD8^+^ T cell-mediated cytotoxicity (Fig 4C and 4D). Together, we found that *M*. *leprae* infection had significantly altered the antigen-presenting function of macrophages. Collectively, live *M*. *leprae*-infected macrophages preferentially primed Treg-type responses, with reduced Th1-type and cytotoxic T cell function, compared to the killed *M*. *leprae*-treated macrophages.

### Macrophages in *L-lep* patients preferentially expressed M2-type cytokines in vivo

**Fig 5:**
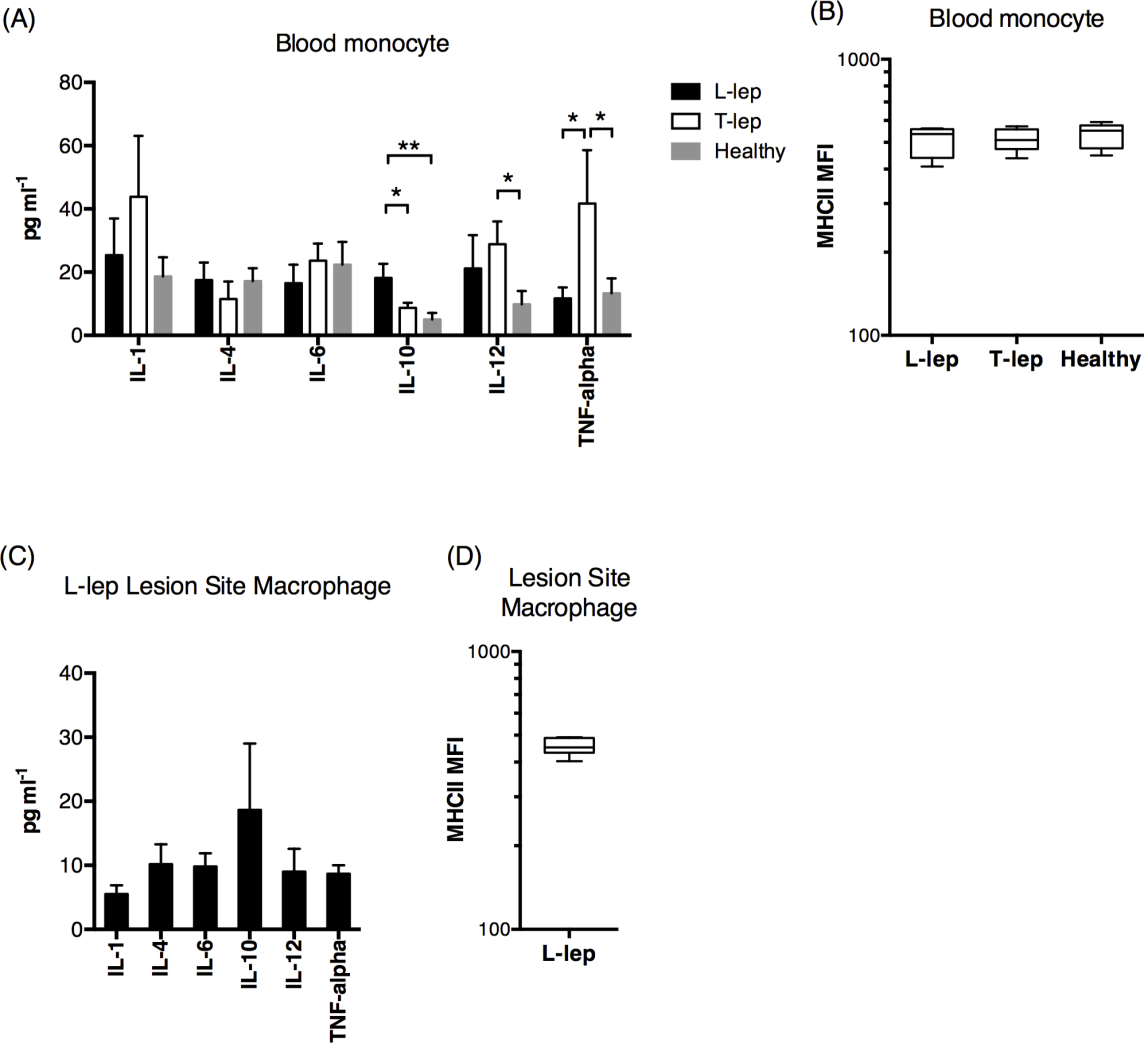
Peripheral blood monocytes and lesion site macrophages in L-lep patients exhibited high IL-10 and low TNF-alpha and MHC class II expressions. (A) The cytokine expression profile by peripheral blood monocytes from L-lep (N = 13) and T-lep (N = 12) patients, as well as healthy volunteers (N = 6) following a 6-day incubation. (B) The MHC class II expression of peripheral blood monocytes from L-lep and T-lep patients and healthy volunteers. (C) The cytokine expression profile by lesion site macrophage from L-lep (N = 7) patients, following a 6-day incubation. (D) The MHC class II molecule expression of lesion site macrophages L-lep patients. *: p < 0.05.

To assess the relevance of our findings in vivo, we examined the peripheral blood monocytes in typical T-lep and L-lep patients, and found that monocytes from L-lep patients preferentially expressed IL-10 while those from T-lep patients preferentially expressed IL-12 and TNF-alpha, compared to healthy individuals (Fig 5A). No significant differences in MHC class II expression between L-lep and T-lep patients were found (Fig 5B). We also examined the leprosy tissue lesion site macrophages from a subset of L-lep subjects who agreed to provide skin lesion samples. IL-10 was the most highly expressed cytokine in L-lep lesion macrophages, which also presented low MHC class II expression (Fig 5C and 5D). We were unable to obtain sufficient numbers of lesion macrophages from T-lep patients due to small lesion site.

## Discussion

In this study, we presented a comparative analyses of live vs. killed *M*. *leprae* in stimulation of macrophages. Live *M*. *leprae*-infected macrophages had significantly higher IL-10 and CD163 expression and lower MHC class II expression, whereas killed *M*. *leprae*-treated macrophages had significantly higher IL-1beta, IL-12 and TNF-alpha, compared to untreated macrophages, demonstrating a difference in the polarization of macrophages by live and killed *M*. *leprae*. Furthermore, this difference in polarization was preserved even after the removal of live *M*. *leprae* and restimulation with killed *M*. *leprae*, suggesting that the differentiation toward M2 differentiation was committed. Previous studies treating whole PBMCs with live BCG vaccine have found that live BCG vaccines induced regulatory T cell phenotype and function[4,16]. Here, by treating PBMC components separately, we found that *M*. *leprae*, live or killed, did not act on naive T cells directly. Rather, through treatment of macrophages with live or killed *M*. *leprae*, different types of T cell responses were primed. Live *M*. *leprae*-treated macrophages preferentially induced regulatory T cell phenotypes and resulted in reduced CD8^+^ T cell cytotoxicity. Finally, these trends were conserved in leprosy patients, especially in the lesion site macrophages.

Enrichment of M2 genes and FoxP3^+^ T cells were previously demonstrated L-lep patients[6,14,15], but it was not clear whether immunosuppressed patients and individuals with a more regulatory immune status tended to develop a more severe leprosy disease, or whether *M*. *leprae* could actively subvert the immune response toward a more regulatory type. Our data showed that the latter scenario was possible by demonstrating that *M*. *leprae* infection could directly initiate immunoregulatory responses and result in suppressed cytotoxicity, in monocytes derived from healthy uninfected individuals, thus providing mechanistic insight on the regulation of adaptive immune responses in leprosy. We in addition demonstrated that live *M*. *leprae*-infected macrophages were more resistant to CD8^+^ T cell-mediated cytotoxicity, potentially contributing to the persistence of *M*. *leprae* in the infected subject. Interestingly, the specific type of antigen being used for stimulating macrophages had a profound impact on the final outcome of the induced T cell responses. Macrophages primed by killed *M*. *leprae* did not exhibit high regulatory activity, and preferentially primed Th1-type responses. Our study highlighted the important contribution of different types of macrophages in modulating adaptive T cell responses. The results emphasized the possibility that *M*. *leprae* may utilize infected macrophages as a pathogen evasion strategy.

Our results also raised many questions that require further examination. To avoid potential cross-reactivity from previous BCG vaccination of our study cohort, we limited our examination on CD45RA^+^ naive T cells. Whether live infected-macrophages could alter memory T cell responses would require further study. In examining anti-*M*. *leprae* cytotoxicity, we focused on CD8^+^ T cells. It was also shown that the lysis of *M*. *leprae*-pulsed macrophage was abolished by 96.5% with the addition of anti-CD4 antibody and 80% with anti-HLA-DR antibody, compared to 55% with anti-CD8 antibody[25], suggesting an essential role of CD4^+^ T cell-mediated cytotoxicity and/or T cell help in the clearance of *M*. *leprae* infection. Combined with the downregulation of MHC class II molecules on live *M*. *leprae*-infected macrophages, the impact of live or killed *M*. *leprae* on CD4^+^ T cell immunity requires further studies. The observation that restimulating live *M*. *leprae*-infected macrophages with killed *M*. *leprae* seemed to augment, rather than reverse, the IL-10 production also needs mechanistic explanations. Previously, IL-10 was shown to inhibit the production of multiple cytokines by dendritic cells after Toll-like receptor (TLR) activation through the negative regulation of MyD88-dependent signaling[26–28]. Therefore, it is possible that the initial IL-10 production by live *M*. *leprae* infection had subverted TLR activation by Gram-positive bacterial motifs in these macrophages, which requires experimental examinations.
